# Nanoscale Examination of Microdamage in Sheep Cortical Bone Using Synchrotron Radiation Transmission X-Ray Microscopy

**DOI:** 10.1371/journal.pone.0057942

**Published:** 2013-03-05

**Authors:** Garry R. Brock, Grace Kim, Anthony R. Ingraffea, Joy C. Andrews, Piero Pianetta, Marjolein C. H. van der Meulen

**Affiliations:** 1 Sibley School of Mechanical and Aerospace Engineering, Cornell University, Ithaca, New York, United States of America; 2 School of Civil and Environmental Engineering, Cornell University, Ithaca, New York, United States of America; 3 Stanford Synchrotron Radiation Lightsource, Menlo Park, California, United States of America; 4 Research Division, Hospital for Special Surgery, New York, New York, United States of America; University of Notre Dame, United States of America

## Abstract

Microdamage occurs in bone through repeated and excessive loading. Accumulation of microdamage weakens bone, leading to a loss of strength, stiffness and energy dissipation in the tissue. Imaging techniques used to examine microdamage have typically been limited to the microscale. In the current study microdamage was examined at the nanoscale using transmission x-ray microscopy with an x-ray negative stain, lead-uranyl acetate. Microdamage was generated in notched and unnotched beams of sheep cortical bone (2×2×20 mm), with monotonic and fatigue loading. Bulk sections were removed from beams and stained with lead-uranyl acetate to identify microdamage. Samples were sectioned to 50 microns and imaged using transmission x-ray microscopy producing projection images of microdamage with nanoscale resolution. Staining indicated microdamage occurred in both the tensile and compressive regions. A comparison between monotonic and fatigue loading indicated a statistically significant greater amount of stain present in fatigue loaded sections. Microdamage occurred in three forms: staining to existing bone structures, cross hatch damage and a single crack extending from the notch tip. Comparison to microcomputed tomography demonstrated differences in damage morphology and total damage between the microscale and nanoscale. This method has future applications for understanding the underlying mechanisms for microdamage formation as well as three-dimensional nanoscale examination of microdamage.

## Introduction

Bone tissue has a load bearing hierarchical structure comprised of many levels [1] in which damage occurs through activities of daily living [2], reducing the strength, stiffness and energy dissipation of the whole bone [3]. This damage, referred to as microdamage, typically consists of small cracks or bone structure damage that are subsequently repaired through remodeling. Microdamage often occurs around lacunae, and the osteocytes within the lacunae are thought to signal the remodeling process when microcracks occur [4], [5]. Examination of these mechanisms is difficult given that the majority of visualization techniques have resolutions that do not resolve the edges of lacunae and canaliculi. Current nanoscale methods only visualize damage on surfaces [6]. A method for microdamage visualization at the nanoscale with a larger depth of focus would be useful for further examining damage mechanisms and remodeling processes.

Visualization of microdamage is typically accomplished through fluorochrome, basic fuchsin or x-ray negative staining [6]–[11]. With these staining techniques, microcracks are visualized as 2D discrete cracks, diffuse damage or cross-hatching. Discrete microcracks typically occur in interstitial bone and are larger than canaliculi but smaller than the vascular canals [12]. Diffuse damage is characterized as a large region of staining in which cracks may or may not be apparent at the scale of observation [13]. Cross-hatch microdamage involves patterned cracks occurring in the bone [14].

Uranyl acetate and barium sulfate have been used as x-ray negative stains (i.e. with high x-ray absorption) for microdamage visualization in bone [6]; [15]–[18]. Microdamage visualization using these methods has been completed with SEM and micro-CT; however, limitations exist for the region viewed and the resolution. SEM can image at the nanoscale but is only able to visualize the surface material of a sample. Micro-CT typically uses voxel sizes of 10 µm or larger [15]–[18]. Methods have also been developed with stains such as gold nanoparticles [19] or terbium nanoparticles [20] to gain higher resolution and view damage at a smaller scale. Confocal microscopy has also been used on fluorochrome-stained slices to visualize microdamage in three dimensions [21].

**Figure 1:**
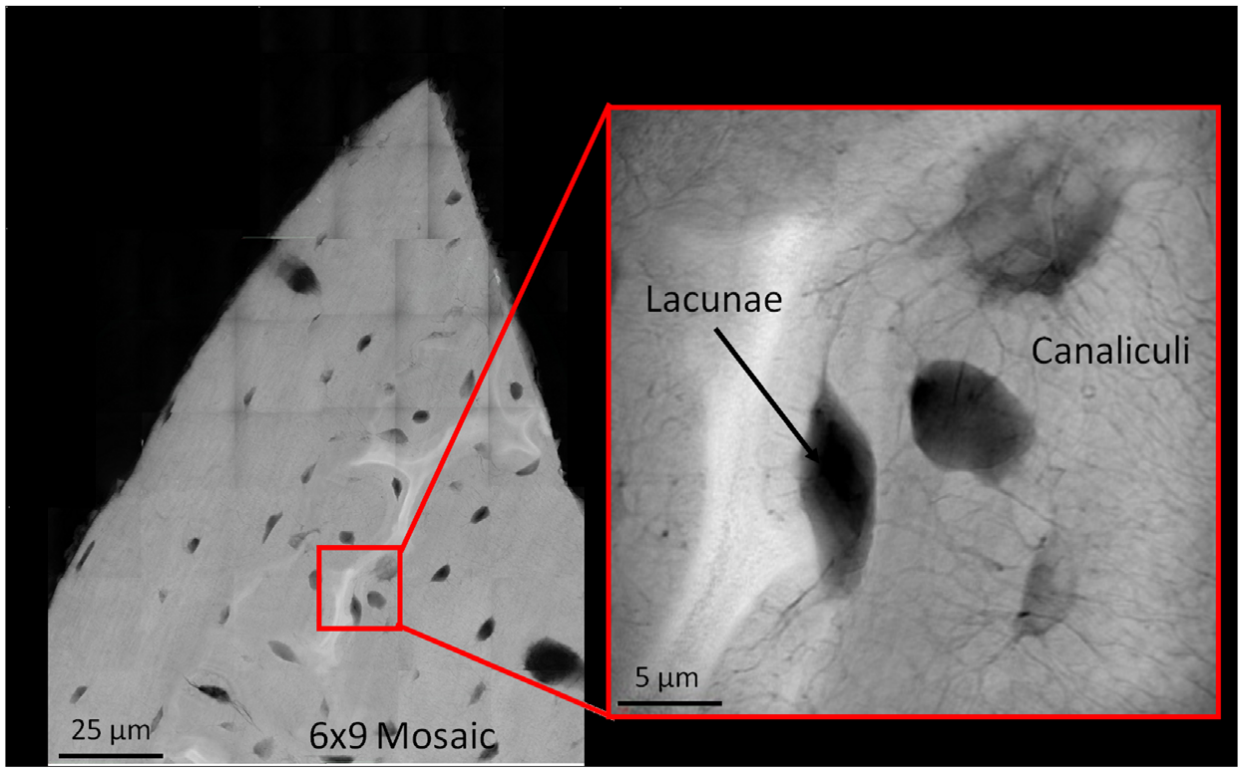
Representative transmission x-ray microscopy images of cortical bone. Representative TXM absorption contrast image (acquired at 7.1 keV) illustrating lacunae and canaliculi present in rat cortical bone (slices 50 microns thick) with grey areas indicating bone, and black areas indicating background, lacunae and canaliculi. Left figure, 6×9 mosaic of low resolution images; right figure, single high resolution image of region. No staining is present in this image; grey-scale variation represents attenuation differences in the tissue.

Transmission X-Ray Microscopy (TXM) uses monochromatic hard x-rays from synchrotron radiation to create x-ray transmission and absorption images with a resolution of 30 nanometers [22]. Bone features, such as osteocyte lacunae and canalicular networks, are visible with TXM (Figure 1). Tomography can also be acquired on small volumes with nanoscale voxel sizes. Stains with strong x-ray absorption can be used to indicate damage by binding to damage present in bone. Differences in attenuation can indicate damage and differentiate stained microdamage from bone and its structures.

This paper describes a method for nanoscale visualization of microdamage in cortical bone tissue using x-ray negative staining and synchrotron-based x-ray imaging. After staining bone with lead-uranyl acetate, samples were imaged using TXM. Analysis of sections loaded in three- and four- point bending demonstrated the sensitivity of the method to detect differences between monotonic and fatigue loading. This method has many future applications for visualization of damage at the nanoscale, leading to increased knowledge about skeletal damage mechanisms.

## Materials and Methods

Three sets of cortical bone beams with different applied loading were used to examine microdamage: (Set 1) intact beams loaded in three point bending, (Set 2) intact beams loaded in four point bending fatigue, and (Set 3) notched beams loaded in three point bending. For all beams, three millimeter wide sections of bone were removed from the cranial portion of the diaphysis of sheep femurs using a low speed diamond saw (Buehler Corp., Lake Bluff, IL). The femurs were obtained from skeletally mature sheep used in a study approved by the Colorado State University Institutional Animal Care and Use Committee. Sheep of this age have previously been shown to have mainly plexiform cortical bone [23]. Sections were then polished using a grinding wheel at 200 rpm using 15, 5 and 1 micron lapping films with ethylene glycol to a final dimension of 2×2×20 mm. Ethylene glycol was used to lubricate between the sample and lapping film to prevent mineral leaching [24].

Beams were loaded monotonically or in fatigue to produce different quantities of damage. The first set of beams (n = 13) was loaded in three-point bending with a preload of 5 N to ensure contact with the sample followed by a 0.25 mm displacement (approx. equivalent to 135 MPa maximum normal stress, 7,500 µε maximum normal strain). Monotonically loaded un-notched beams were used to examine whether microdamage from a single loading cycle could be viewed with TXM. The second set of beams (n = 26) was loaded in four-point bending fatigue from 4.8 to 48 N (approx. equivalent to 10 to 90 MPa maximum normal stress, 555 to 5,000 µε maximum normal strain) at 2 Hz for 20,000 cycles. Beams were tested in hydroxyapatite buffered saline at room temperature. Fatigue loaded beams were used to examine microdamage formed through repetitive loading. The third set of beams (n = 23) had a 200 micron deep notch applied to the center of the periosteal side using a razor blade. Samples were then loaded monotonically in three-point bending to a force of 25 N (approx. equivalent to 94 MPa maximum normal stress, 5,200 µε maximum normal strain) such that the notched side was in tension. Notched samples were used to localize damage to a specific region to compare damage morphologies. The entire notch region was considered the tensile region and was not further subdivided, while the compressive region was the side opposite of the notch. Differing number of samples in each set was due to acquisition time with the TXM and limited availability of the instrument. Statistical comparisons accounted for these differences.

Previously established procedures for uranyl acetate staining with micro-CT and SEM were used to indicate microdamage after loading [6], [16], [25]. Bulk sections of length 5 mm were removed from the center of the beam and stained in a solution of equal parts 8% uranyl acetate in 70% acetone and 20% lead (II) acetate in 70% acetone for one week. After one week the samples were placed in a 1% ammonium sulfide in 70% acetone solution for one week with the solution changed at 3 days. Post staining, thick sections were removed and polished using a precision grinding wheel (Allied High Tech, Rancho Dominguez, CA) to approximately 50 micron thickness using 15, 5, and 1 micron lapping films with ethylene glycol as a lubricant. Sections of monotonically-loaded unnotched beams (Set 1) were created at the point of loading for samples in the region of highest loading in the transverse plane of the bone. Fatigue sections (Set 2) were taken from the center of the beam in the region of highest load and created in both the transverse and longitudinal planes of the bone. Sections of notched beams (Set 3) were taken longitudinally to view localized damage around the notch. This orientation was perpendicular to the direction examined in the unnotched samples and aligned with the axis of the osteons to better capture regions of damage with loading.

Samples were imaged at the Stanford Synchrotron Radiation Lightsource (Menlo Park, CA) using the Transmission X-ray Microscope (Beamline 6-2c, Xradia; Pleasanton, CA). Absorption images were acquired with an energy of 7.1 keV (2048×2048 pixels, 10.4 nm (monotonic loading) or 13.98 nm (fatigue loading) pixel size, 1 sec exposure). Images were taken along the axis of loading in the compressive, tensile and neutral axis regions of the cortical bone beams. To characterize tissue morphology and staining, both low resolution and high resolution images were taken. Low resolution images used frame averaging of two images for each field of view. Neighboring images were stitched, aligned and smoothed to create larger mosaics (11×11 tiles) of each region [26]. High resolution images were taken in areas with microdamage by frame averaging 10 separate images of the same area to form a single image. Analysis to examine microdamage was then performed on the low resolution mosaics and high resolution.

Comparisons were made between monotonic and fatigue loaded unnotched specimens through damage quantification. Regions corresponding to tensile and compressive normal stresses were identified based on the continuum level loading applied to the beams. Staining was identified and quantified for these tensile and compressive regions. First, to threshold uranyl acetate from bone each pixel in every large mosaic was counted and binned using a histogram with a thousand bins (Matlab, Mathworks, Natick, MA). A triangle method was used to threshold the bone from the uranyl acetate [27]. This method involves representing the image data as a histogram with a Gaussian peak representing the attenuation of the bone and a higher attenuation tail representing uranyl acetate. Total counts for uranyl acetate and bone were then summed, and the lead-uranyl acetate counts were normalized by the total bone counts. Stain totals in monotonically loaded samples were compared to fatigue loaded samples using a Student’s t-test (JMP, SAS, Cary NC). This comparison was only completed on images in the tensile and compressive regions. The lack of staining in the neutral axis region with monotonic loading precluded statistical comparisons. Damage was expected at the neutral axis only in the fatigue samples, given the change in the position of the neutral axis as damage accumulates, because the modulus changes differently in the tensile and compressive regions.

To compare this new approach with a more established technique, notched sections were imaged by microcomputed tomography (micro-CT) prior to imaging with TXM. Sections were imaged at a 3.5 micron voxel size with micro-CT (Scanco Medical µCT 35 system, Scanco Medical, Brüttisellen Switzerland). Images were thresholded to separate bone from background using a linear attenuation coefficient (μ, 1/cm) value of 2.5. The lead-uranyl acetate (UA) staining was then thresholded from the bone using an attenuation coefficient value of 6. Images were rotated such that an edge view was present, and the UA staining was overlaid to create a 2D micro-CT image similar to that of TXM. Corresponding TXM images of the same region were binned to match the voxel size used for micro-CT (MATLAB, Mathworks, Natick, MA). Thresholds were used to separate the background, bone and UA by placing the binned pixels into a histogram and fitting the peaks for background, bone and UA. Given the small field of view with TXM, no measures could be calculated with micro-CT; however, observations about damage morphology and distribution were drawn through image visualization and qualitative comparison.

## Results

**Figure 2:**
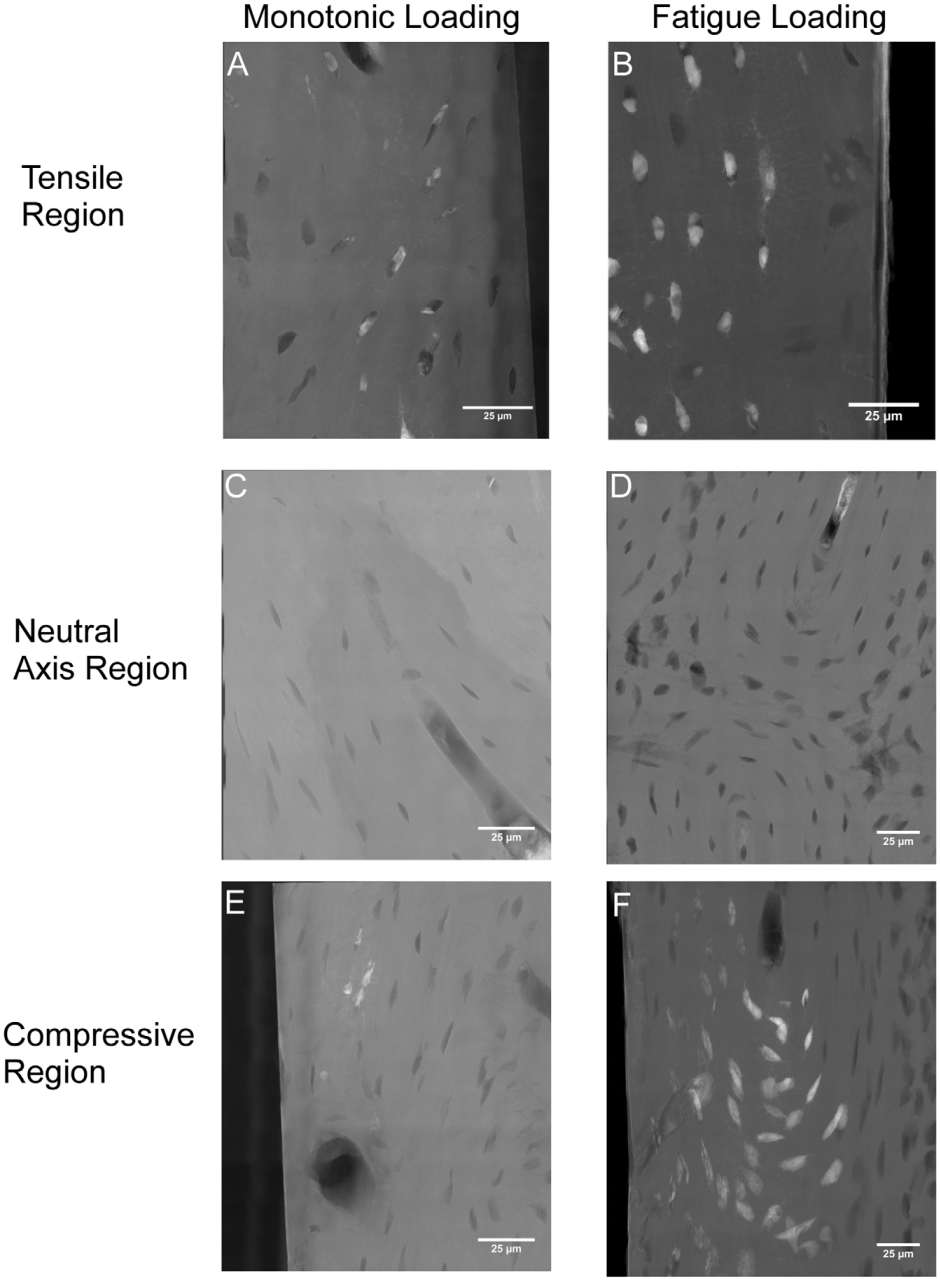
Lead-Uranyl Acetate staining following monotonic and fatigue loading of cortical bone beams. Staining of the tensile (A), neutral axis (C), and compressive (E) regions of monotonically loaded samples. Staining of the tensile (B), neutral axis (D), and compressive (F) regions of fatigue loaded samples. TXM images shown in transmission mode; white indicates high attenuating lead-uranyl acetate staining, grey indicates bone, and black indicates background. All scale bars are 25 microns.

**Figure 3:**
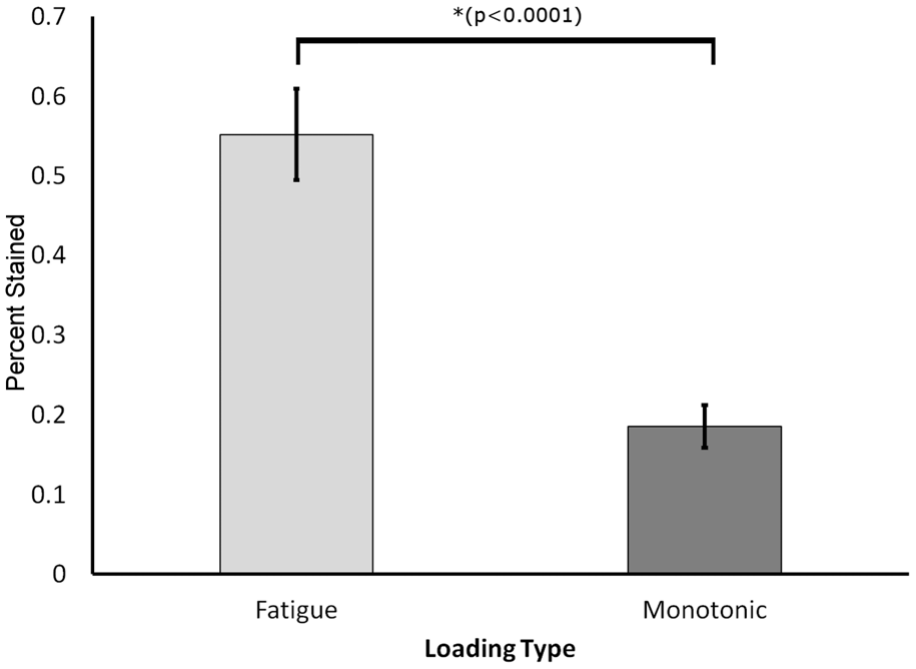
Increased stain uptake in fatigue-loaded cortical bone samples. Summary of total staining in TXM images for cortical bone sections following fatigue and monotonic loading with both compressive and tensile regions pooled. Fatigue loading produced significantly more stain, indicating repeated loading creates greater damage formation allowing for increased uptake of the stain into bone tissue.

Unnotched samples from both monotonic and fatigue loading demonstrated the ability of TXM to image microdamage. Unnotched samples with a monotonic load (Set 1) had staining of bone structures. Staining was typically localized to areas of maximum stress and not present at the neutral axis. Staining was solely present in existing bone structures such as lacunae and canaliculi; no stain was evident in tissue outside of bone structures in either the tensile and compressive regions. For samples with fatigue loading (Set 2) staining of the bone tissue was present in existing bone structures in all samples (26 of 26 samples imaged) and to new surfaces outside of bone structures in some samples (6 of 26 samples imaged). Repetitive loading increased the amount of stain present in the bone tissue compared to monotonic loading (Figure 2). The percent stained tissue area was greater (p<0.0001) in the fatigue loaded sections as compared to the monotonically loaded sections (Figure 3). Greater stain presence in fatigue loaded sections illustrated that lead-uranyl acetate staining is an indicator of microdamage presence in bone, as more damage would be expected to occur in repetitively loaded tissue.

**Figure 4:**
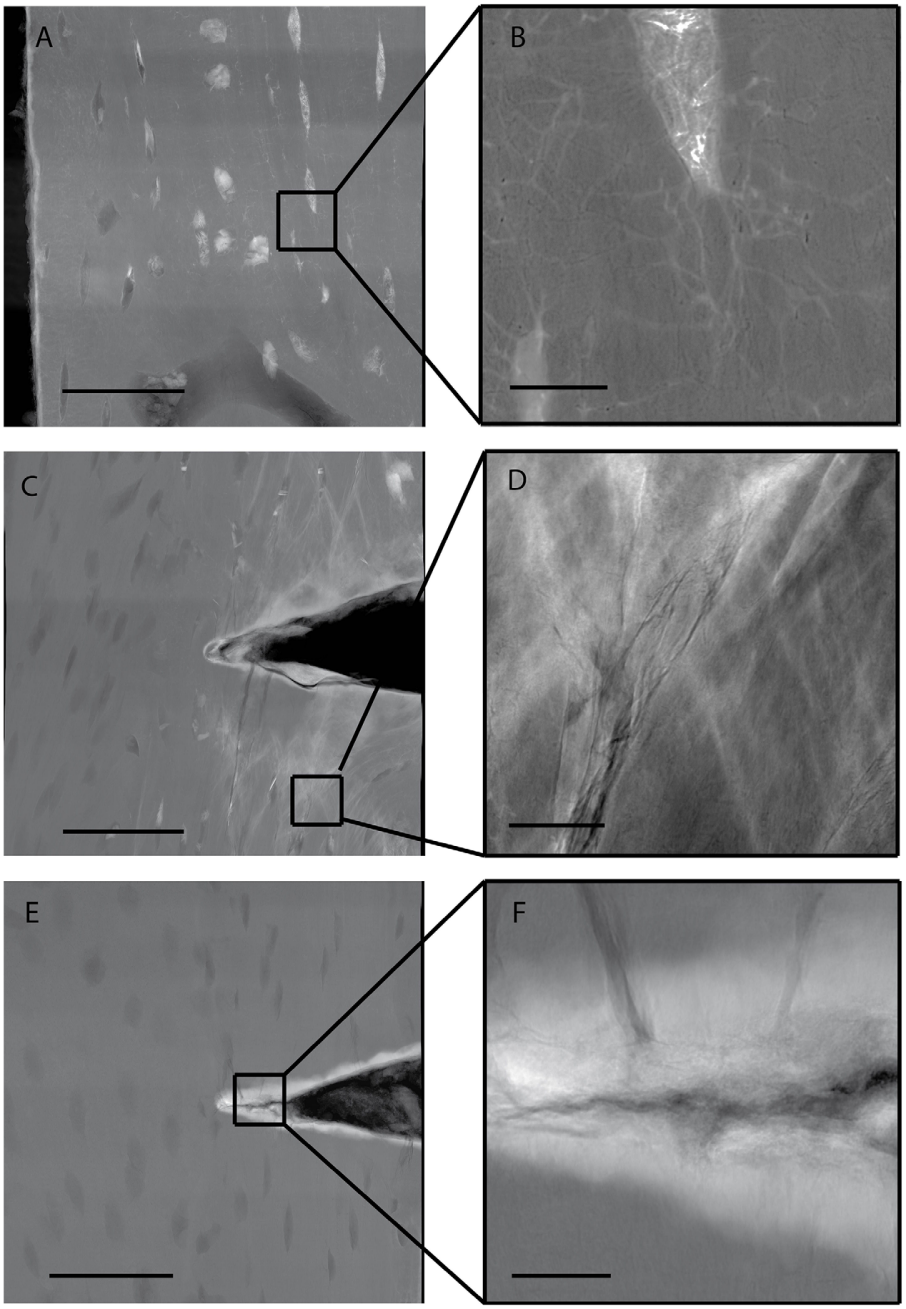
Lead-Uranyl Acetate staining of damage morphologies in notched bone samples. (A, B) Staining of lacunae and canaliculi in the compressive region seen in 20 of the 23 samples; (C, D) Cross hatching damage around notch tip in the tensile region observed in 10 of 23 samples; (E, F) Crack propagating from notch tip in the tensile region in a single sample. Staining appears white due to high attenuation of lead-uranyl acetate, with bone tissue appearing grey and voids black. Scale bar: A,C,E = 50 µm; B,D,F = 5 µm. Sample created in the longitudinal plane of the bone.

**Figure 5:**
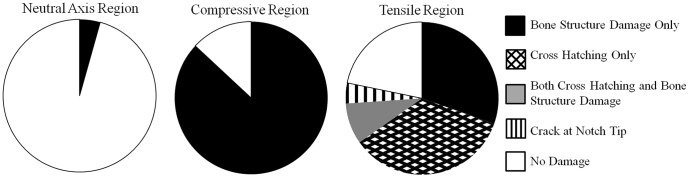
Classification of damage morphologies imaged in notched bone specimens. Summary of damage morphologies observed in the notched samples in each loading region (n = 23 samples total). The majority of samples had staining of bone structures in the compressive region, and damage occurred at the neutral axis in only one sample. The tensile microdamage was mainly staining of bone structures or cross hatching in the notched samples with a single sample having a propagated crack. Damage in the unnotched samples consisted only of bone structure staining.

Microdamage in notched samples (Set 3) occurred in three forms: staining of existing bone features, cross-hatching damage, and discrete cracks. The most common form was staining of lacunae and canaliculi in the tensile and compressive regions (Figure 4 A, B; Figure 5). Cross-hatching microdamage occurred around the notch tip in 10 of 23 samples tested (Figure 4 C, D; Figure 5). Samples with cross-hatch damage occasionally also had stained bone structures (lacunae and canaliculi) adjacent to the cross-hatching. A single sample had a crack propagating from the notch tip (Figure 4 E, F; Figure 5). No staining occurred in the neutral axis region in 22 of 23 notched samples (Figure 5).

**Figure 6:**
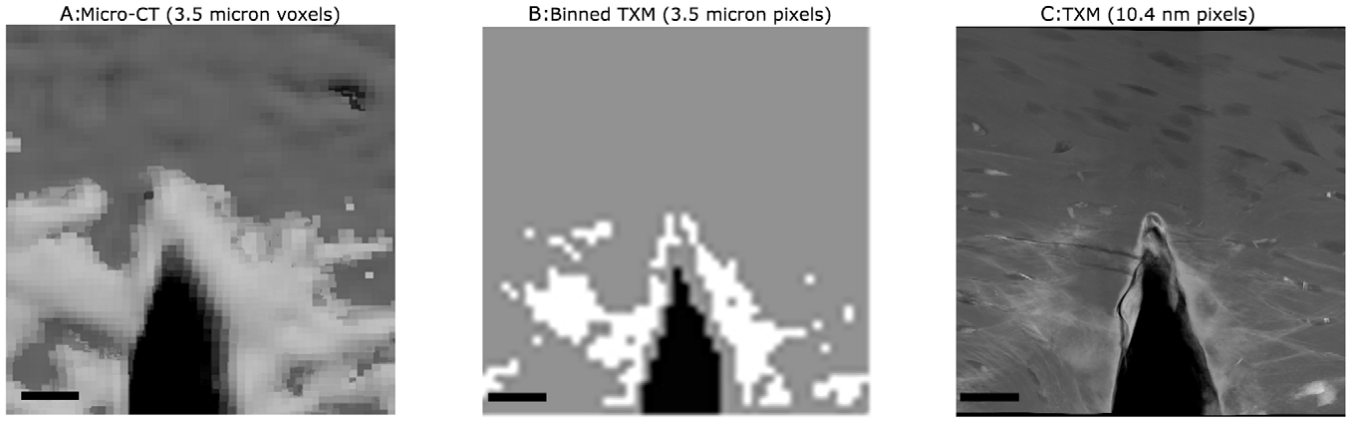
Comparison of damage visualization with TXM compared to micro-CT scanning. Comparison of micro-CT with TXM images of similar region of interest in notched cortical beam samples. In all images white areas indicate high attenuating lead uranyl acetate, grey represents bone and black represents background. First image (A) shows micro-CT scan of staining, second (B) is TXM imaged binned to same pixel size as micro-CT scan, and third (C) is raw TXM image. These images illustrate differences in damage morphology and partial volume effect that occur between micro-CT and TXM. Staining of bone structures and nanoscale damage is not visible using micro-CT; scale bar = 25 microns.

Differences in damage morphology were evident between micro-CT and TXM images. In the micro-CT reconstruction (Figure 6A) staining at the microscale appeared to cover a larger region of the sample due to partial volume effects of the x-ray negative stain. The micro-CT voxels were also larger than the bone structures, that were visible with TXM, and therefore micro-CT was not able to resolve the damage morphologies present in the bone tissue. Binning and thresholding of the TXM image to create pixels at the same scale as the micro-CT voxels (Figure 6B) reduced the stain area from micro-CT. Finally, the localized nature of the stain was evident in the full-scale, high resolution TXM image (Figure 6C).

## Discussion and Conclusions

A method to examine microdamage with nanoscale resolution creating an x-ray projection image is presented using previously published staining techniques [6], [16]. This method demonstrated nanoscale damage morphologies including staining of existing bone features, cross-hatching, and a discrete crack in a single specimen (Figure 4; Figure 5). Lead-uranyl acetate staining occurred in the regions of highest stress where damage would be expected and not in regions of low stress, indicating the lead-uranyl acetate was correlated with the higher stress regions where microdamage would be expected.

Previously microdamage was reported at the neutral axis of bone beams loaded in bending fatigue [13]. Here staining did not occur at the neutral axis of samples with monotonic loading but did occur in the fatigue loaded samples. UA is therefore able to move to the interior of a sample, which may reflect more transport paths in fatigue loaded sections allowing the stain to penetrate further into the interior of the sample. Prior studies have demonstrated different morphologies in compressive and tensile stress regions, with compressive regions having more microcracks and tensile regions having more diffuse damage [13]. Results from this study showed similar morphologies of stained bone structures in both compressive and tensile regions. This difference may reflect that the bone structures visualized here are not visible with techniques used in prior studies. Limitations of the techniques and samples used should be noted for examining differences in the damage evident. Fatigue samples used in this study were loaded to a fixed number of cycles and may not have experienced equal degrees of stiffness loss, contributing to different damage formation. Prior studies focused on osteonal bone whereas plexiform cortical bone was used for this study. Damage in plexiform bovine cortical bone loaded in four point bending fatigue is dominated by viscoplastic creep [28], a limitation of the specimens and loading mechanisms used in this study. Comparisons of the percentage of stained tissue indicated a significantly greater area of staining in the fatigue-loaded samples. If transport of the stain into the bone had solely been by diffusion, differences would not have been present between the monotonic and fatigue loaded samples. Therefore, damage to the tissue must occur to create more paths for the stain to penetrate the tissue. This increased permeability was primarily through the existing lacunar-canalicular network.

Comparison with micro-CT (Figure 6) demonstrated differences in damage morphology and area. Partial volume effects and beam hardening will affect the damage imaged with micro-CT, with the stained region appearing larger than with TXM. Quantification and direct comparison between TXM and micro-CT was not possible due to the limited field of view of TXM. For the equivalent tissue area, micro-CT images contained 2,260 voxels total whereas TXM images contained 250 million pixels. Damage morphologies also appeared different at the different scales with binning and thresholding of the TXM image illustrating similarities between TXM and micro-CT. Damage viewed with micro-CT and x-ray negative stains may, therefore, overestimate the damage regions when damage morphology is below the micro-CT resolution [15]–[18].

This study could not determine the mechanism of increased staining with fatigue loading. Uranyl acetate binds to phosphate groups in cell membranes [29]. The addition of the ammonium sulfide immobilizes the metal, blocking further penetration of the stain. The mechanism of the stain presence is believed to be a combination of binding to hydroxyapatite crystals and space filling within the bone structures. The additional staining in the fatigue loaded specimens likely reflects increased channels caused by damage to the tissue that permit the stain to penetrate further into the bone. Without this additional damage, the stain amounts would be similar for fatigue and monotonic loading, given that all samples were stained post-loading. The uranyl acetate staining may, therefore, be a transport-driven phenomenon, wherein more paths are created with increased loading.

Images viewed in this study differed from prior SEM studies in that samples were viewed as projections sampled through the thickness, rather than surface images [6]. SEM studies are unable to resolve damage more than a few microns below the surface of the sample. TXM allows damage to be viewed throughout a 50 micron thick section of the sample without sputtercoating of surfaces with gold or carbon. While not performed in this study, TXM also has the capability to create a tomography with 10 nm voxels for three dimensional nanoscale microdamage visualization and quantification on samples with depth and widths at or below 50 microns [22]. Lead-uranyl acetate staining has been used previously to create tomographic reconstructions of cancellous bone samples using micro-CT [16]. These tomographies had voxel sizes of 10 micrometers, in comparison to voxel sizes of 10.4 nanometers with TXM. Synchrotron micro-CT without staining has been used for three-dimensional visualization of microdamage and cracks in human cancellous bone using a 1.4 micrometer linear voxel size [30]. Synchrotron radiation micro-CT has also been used to examine crack propagation in murine cortical bone with a 700 nm resolution [31]. TXM with lead-uranyl acetate stain has the potential to build on this knowledge by creating three-dimensional reconstructions of microdamage with 10 nm voxels.

Studies in cortical bone demonstrated that damage will arrest at or move along cement lines or lamellae before moving through osteons [32]–[34]. Damage in the current study occurred in structures around and along lamella in the bone tissue, consistent with regions in which damage is typically observed at the microscale [35]. The low level of loading applied in the notched samples may account for only a single sample having a crack propagate from the notch. The damage observed may, therefore, be a precursor to larger discrete cracks. Diffuse damage in fatigue loaded, notched samples was believed to be damage to bone features during the early stages of the damage process; however, these features were not visible with fluorochrome staining and the image resolution used [36]. Resolution limits associated with confocal microscopy prevented the determination of whether microcracks penetrated lacunar walls in a study examining microdamage around osteocyte lacunae in tensile loaded cortical bone [4]. TXM visualized not only osteocyte lacunae, but also damage to the canaliculi, allowing for an understanding of the role of nanoscale bone structures in microdamage [22].

TXM for microdamage analysis has several important limitations. The sample must be no more than 50 microns thick and have a smooth surface. Therefore, sample preparation is time consuming and intensive. For tomographies, x-ray paths through the sample are limited to 50 microns for all vectors through the sample. Recent advances also allow for larger samples to be imaged using an extended depth of focus [37]. Viewing large areas requires stitching of individual images to create mosaics which are computationally intensive as well as time consuming to acquire. Finally, even when images are combined, the total field of view with a mosaic is small, on the order of hundredths of a square millimeter. Given the small areas viewed, quantification of tissue properties and microdamage analysis may be difficult to generalize.

To our knowledge, TXM with lead-uranyl acetate staining is the first method to create a projection image of microdamage in bone at the nanoscale. Staining occurred primarily in bone structures in the lacunar canalicular network, not on new surfaces, regions below the resolutions of other microdamage imaging modalities. Nano-CT using synchrotron x-rays of damaged samples and examination of additional loading conditions will allow for a greater understanding of microdamage mechanisms and precursors.
